# Metabolic restraining of epigenetic modifications promotes cardiomyocyte proliferation

**DOI:** 10.1093/lifemeta/load047

**Published:** 2023-12-01

**Authors:** Xiuxiu Liu, Bin Zhou

**Affiliations:** New Cornerstone Science Laboratory, State Key Laboratory of Cell Biology, CAS Center for Excellence in Molecular Cell Science, Shanghai Institute of Biochemistry and Cell Biology, Chinese Academy of Sciences, University of Chinese Academy of Sciences, Shanghai 200031, China; New Cornerstone Science Laboratory, State Key Laboratory of Cell Biology, CAS Center for Excellence in Molecular Cell Science, Shanghai Institute of Biochemistry and Cell Biology, Chinese Academy of Sciences, University of Chinese Academy of Sciences, Shanghai 200031, China; Key Laboratory of Systems Health Science of Zhejiang Province, School of Life Science, Hangzhou Institute for Advanced Study, University of Chinese Academy of Sciences, Hangzhou, Zhejiang 310024, China; School of Life Science and Technology, ShanghaiTech University, Shanghai 201210, China

**The metabolic state of a cell is closely related to its structure and function in adult mammalian cardiomyocytes. These adult cardiomyocytes primarily use fatty acids as an energy substrate to support heart contraction. Recently, Li and his colleagues reported that inhibiting fatty acid oxidation in cardiomyocytes keeps them in an immature state. This influences epigenomic modifications and ultimately increases the proliferation capacity of the cardiomyocytes.**

Heart failure, caused by excessive loss of beating cardiomyocytes, is one of the leading causes of death worldwide [1]. Numerous stem cell studies have attempted to identify a source of new cardiomyocytes in adult mammals. However, the results predominantly suggest that there are no cardiac stem cells available to replenish the loss of cardiomyocytes following heart failure [2]. The proliferative capacity of mammalian cardiomyocytes rapidly declines after birth, reaching a very low level in adulthood [3, 4]. Adult cardiomyocytes lack sufficient proliferative capacity to compensate for the loss of cardiomyocytes after cardiac injury, which results in scar tissue formation. Recent studies have, therefore, focused on stimulating the proliferation of adult cardiomyocytes. Approaches include identifying combinations of small molecules [5] and inducing cell-cycle genes in non-regenerating cardiomyocytes [6]. Other mechanisms, such as DNA damage and hypoxia [7], may also play a role in the regenerative processes. Studying the differences between zebrafish and mammals, and disparities between newborns and adults could also help in recapitulating the regenerative mechanisms in adult mammal cardiomyocytes and overcoming the proliferation barrier of these cardiomyocytes.

Cardiomyocytes in neonates and adults differ in two major ways: proliferative capacity and energy metabolism. Aided by the enrichment of oxygen content and the transition of energy metabolism from glycolysis to fatty acid β-oxidation, the turnover rate of cardiomyocytes rapidly decreases. The heart’s fuel must be continuously supplied by the blood, as cardiomyocytes cannot store glycogen. As a result, these cells require long-term stable energy substrates. Terminally differentiated cardiomyocytes, serving as a high energy-demand cell type, primarily produce energy through oxidative phosphorylation in the mitochondria and glycolysis in the cytoplasm. However, mitochondrial oxidative phosphorylation accounts for 95% of the total energy production [8]. Nicotinamide adenine dinucleotide (NADH), essential for oxidative phosphorylation, is primarily generated in the mitochondrial matrix by the Krebs cycle. In mammalian hearts, acetyl coenzyme A (acetyl-CoA), the main substrate for the Krebs cycle, primarily originates from fatty acids, lactate, glucose, ketones, and amino acids (Fig. 1).

**Figure 1:**
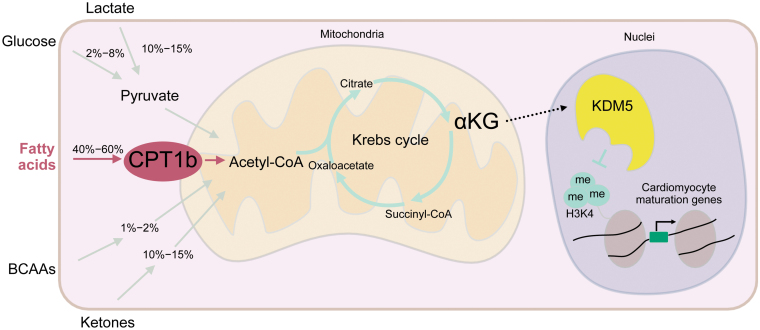
Different energy substrates contribute to the cellular amount of acetyl-CoA and participate in the ATP production of cardiomyocytes through the Krebs cycle. Inactivation of CPT1b impaired fatty acid oxidation and further caused accumulation of αKG. Accumulation of αKG further erases the H3K4me3 modifications on cardiac maturation genes through demethylase KDM5, thus leading the cardiomyocytes to an immature state and proliferative state. BCAAs, branched-chain amino acids.

Among the various energy substrates, fatty acids are the main fuel in cardiac muscle. They are β-oxidized to generate a large amount of acetyl-CoA, which participates in the Krebs cycle and ultimately enters into mitochondrial oxidative phosphorylation to generate ATP. Several previous studies have focused on modified energy supply and heart regeneration [9, 10]. However, the underlying mechanisms of how these metabolic changes control cycling genes remain unknown.

The most efficient way to induce changes in cardiac energy metabolism is by influencing the key enzymes involved in fatty acid oxidation. Long-chain fatty acids in the cytoplasm are first catalyzed by carnitine palmitoyltransferase I (CPT1) to form long-chain acylcarnitine. This is then transferred to the mitochondrial matrix and catalyzed by CPT2 to regenerate fatty acyl-CoA. CPT1 is the rate-limiting enzyme in this process. A recent study by Li *et al*. [11] demonstrated that the proliferation potential of adult cardiomyocytes could be enhanced by inhibiting fatty acid oxidation through the knockdown of *Cpt1b* (the muscle-specific isoform of CPT1). Similar to previous studies, they initially sought to identify differentially expressed genes between neonates and adults. Fortunately, they identified the differentially expressed muscle metabolic gene *Cpt1b*, which is related to fatty acid transportation.

Previous work demonstrated that fatty acid β-oxidation was related to cardiomyocyte proliferation. Hence, the authors wanted to investigate the role of CPT1b in cardiac regeneration and maturation, as its increased expression coincided with cardiomyocyte maturation. By knocking out *Cpt1b* using a constitutive cardiomyocyte Cre mouse line (*αMHC-Cre*) or a tamoxifen-inducible line (*αMHC-MerCreMer*), they observed larger heart sizes and more proliferating cardiomyocytes (stained with proliferation markers like 5-ethynyl-2ʹ-deoxyuridine [EdU], phospho-histone H3 [pH3], or Aurora B) compared to the control mouse hearts. Regarding the size of each cardiomyocyte, the authors discovered that *Cpt1b* knockout (KO) significantly increased the number of the smallest and largest cardiomyocytes. This suggests that *Cpt1b* KO promoted hyperplastic or hypertrophic growth by increasing cardiomyocyte proliferation or their size. The authors further confirmed that the enhanced proliferation of cardiomyocytes also promoted heart regeneration after ischemia–reperfusion (I–R) injury. Knocking out *Cpt1b* from the embryonic stage or at the adult stage both reduced the scar area, cell death, and cardiomyocyte size surrounding the infarct area and enhanced the capacity for pre-existing cardiomyocytes to re-enter the cell cycle and improve cardiac contraction capacity.

A concern is that alterations of the energy substrate could potentially harm cardiac function. It raises the question of whether *Cpt1b* KO stimulated cardiac protective procedures to enhance cardiac function, rather than primarily promoting proliferation. The authors confirmed that signs of improved cardiac function appeared before the reduction of CPT1b protein expression levels. This suggests that cardiac protection impacts cardiac function only after cardiomyocyte proliferation. The authors then sought to understand the changes in cardiomyocytes after *Cpt1b* KO at an overall level. Through RNA-seq analysis of cardiomyocytes, they discovered that *Cpt1b* KO upregulated several signaling pathways related to cell cycle progression and cardiomyocyte dedifferentiation, while downregulating genes associated with cardiomyocyte maturation and contraction. The downregulated genes suggested that the *Cpt1b* KO cardiomyocytes were in a less mature state. The authors further confirmed the immaturity of these cardiomyocytes by detecting mature marker genes (solute carrier family 2 member 4 (*Slc2a4*) and lactate dehydrogenase A (*Ldha*)) and dedifferentiation marker genes (natriuretic peptide precursor A (*Nppa*) and skeletal muscle α-actin gene (*Acta1*)), respectively. Moreover, the reduced γH2A.X^+^ cardiac nuclei and increased expression levels of hypoxia-inducible factor-1α (Hif-1α) in *Cpt1b* KO mouse hearts also suggested that alterations in fatty acid oxidation reduced DNA damage. This reduction may promote cardiomyocyte proliferation.

A healthy heart generates very large amounts of ATP to meet contraction needs. The energy substrates for fuels are flexible, and cardiomyocytes can shift between the main energy substrates to sustain ATP production [8]. Knocking out *Cpt1b* would undoubtedly influence fatty acid oxidation, but how does it affect other metabolic pathways? The authors addressed this by using metabolic flux and Seahorse assays, demonstrating that the cellular amounts of major components of the Krebs cycle, such as acetyl-CoA, citrate, succinate, and malate, did not change significantly. This indicated that other metabolic pathways stepped in to bridge the gap after the suppression of fatty acid oxidation. Candidate metabolic pathways that can supply the lost acetyl-CoA after blocking fatty acid oxidation include pyruvate (generated by glycolysis or lactate gluconeogenesis, which can be converted to acetyl-CoA), ketone bodies, and branched-chain amino acids (BCAAs). The increased expression level of pyruvate dehydrogenase (E1) alpha subunit gene (PDH1A, mitochondrial, also named as PHE1A) suggested that elevated levels of acetyl-CoA were generated from pyruvate after glycolysis or gluconeogenesis. Similarly, the authors found that markers of BCAA catabolic metabolism like α-ketoisovalerate and α-keto-β-methylvalerate showed a marked elevation. The increased levels of propionyl-CoA and methylmalonyl-CoA also indicated that BCAA catabolism supplied the acetyl-CoA pool. Seahorse assays confirmed that the *Cpt1b* KO cardiomyocytes could utilize pyruvate and BCAA more efficiently. The authors also noted significant changes in α-ketoglutarate (αKG) after *Cpt1b* inhibition, along with increased levels of some isocitrate dehydrogenase family enzymes that catalyze isocitrate to αKG. Cardiomyocyte-specific KO of 2-oxoglutarate dehydrogenase (OGDH) also resulted in elevated αKG levels, suggesting that αKG accumulation may also be due to the absence of OGDH. The enzymatic activity of OGDH did reduce after inactivation of *Cpt1b*.

Next, they focused on αKG, which showed the most prominent changes. The impact of altered metabolic patterns on epigenetic modifications has been a long-standing research focus in the field. Conveniently, αKG, previously identified, happens to be a key factor in histone demethylation. They found that histone H3 lysine-4 trimethylation (H3K4me3) was the directly affected heterochromatin demethylase.

Chromatin immunoprecipitation and sequencing (ChIP-seq) analysis of H3K4me3 identified decreased H3K4me3 modifications of cardiac maturation genes, which was consistent with the previous RNA-seq results. They hypothesized that accumulation of αKG led to a reduction in the H3K4me3 modifications of cardiac maturation genes, resulting in cardiomyocytes being more immature and exhibiting higher proliferation capacity. Indeed, treating cultured neonatal cardiomyocytes with extra αKG or overexpression of enzymes that can increase αKG levels truly enhanced their expression of immature genes and proliferation capacity. Furthermore, the authors treated the cultured cardiomyocytes with an inhibitor of αKG-dependent H3K4 demethylase histone lysine demethylase 5 (KDM5), or a competitive inhibitor of αKG, to prevent the proliferation-promoting process. These experiments further indicated that the proliferation-promoting effect of increased αKG was realized through, or at least partially through, KDM5. The authors next directly overexpressed *Kdm5b* (a member of the *KDM5* gene family) in cultured cardiomyocytes, resulting in increased cardiomyocyte cell cycle activity. Conversely, knocking down *Kdm5b* restored the H3K4me3 modifications of cardiac maturation genes and decreased the pro-proliferative effect.

Finally, the authors linked the pro-proliferative effects of inactivation of fatty acid oxidation to a specific gene, which could serve as a potential pharmacological target.

One of the critical outstanding aspects of this work is the challenge of linking metabolic changes at the overall level to a specific gene. The authors focused on one major metabolite, αKG, which significantly increased in levels after inhibition of fatty acid oxidation compared to control hearts. The elevated αKG led them to investigate the epigenetic modifications of histone lysine methylation, specifically H3K4me3. This led the authors to investigate the specific gene family, KDM5, which is a well-known histone demethylase in human cancer.

In future research, it will be necessary to further demonstrate the role of αKG and KDM5 *in vivo* and clarify the upstream-downstream relationship. If possible, future studies could use *in vivo* genetic tools to induce inactivation of αKG at low doses in certain cardiomyocytes and search for other regulatory genes that operate in a similar manner to KDM5. Numerous emerging studies on metabolic alterations to enhance cardiomyocyte proliferation illustrate the potential of promoting cardiac regeneration through changes in metabolic modalities. However, many investigations lack a specific understanding of how metabolism affects cardiomyocyte proliferation and thus limit further advancements in research related to metabolism and cardiomyocyte proliferation. This work is more innovative in that it combines metabolic alterations with epigenetic modifications, thereby broadening the depth and breadth of exploration. Simultaneously, the introduction of epigenetic modifications poses a challenge for future studies that aim to promote cardiomyocyte proliferation using this gene as a target. Because both metabolism and epigenetics are influential factors that affect the whole body, further exploration is needed to rule out secondary effects when targeting metabolic or epigenetic genes. From this study, many researchers in the field see hope in the epigenetic modification gene *Kdm5b* to promote cardiomyocyte proliferation and facilitate cardiac repair after injury. However, there is still a great need for *in vivo* data on gene-specific overexpression in cardiomyocytes to investigate their pro-proliferative effect, as the *in vivo* environment is more complex and variable than that *in vitro*.

Another intriguing question posed by this work is the relationship between immature cardiomyocytes and cardiomyocyte proliferation. It is unclear whether cardiomyocytes regressing to an immature state precedes or accompanies the initiation of proliferative events. Is every enhanced cardiomyocyte proliferation event achieved by forcing mature cardiomyocytes back to an immature state? If so, the number of immature cardiomyocytes must be kept under control; otherwise, a large number of immature cardiomyocytes could greatly reduce the contractile function of the heart. Blocking fatty acid oxidation from the embryonic stage did not convert a large number of cardiomyocytes to an immature state, as the mouse hearts were of normal size in the adult stage. It is noteworthy that not all the *Cpt1b* KO cardiomyocytes reverted to an immature state. This raises the question of what the underlying mechanism is that controls this regression process.

In summary, the work by Li *et al*. [11] demonstrates that metabolic alterations within cardiomyocytes are closely linked to epigenetic modifications and control the proliferation capacity of cardiomyocytes. This research not only uncovers how metabolism regulates cell proliferation through epigenetic regulation but also raises several new scientific questions and provides numerous opportunities for stimulating heart regeneration.

## References

[1] Tzahor E, Poss KD. Science 2017;356:1035–9. 28596337 10.1126/science.aam5894 PMC5614484

[2] Li Y, He L, Huang X et al. Circulation 2018;138:793–805. 29700121 10.1161/CIRCULATIONAHA.118.034250

[3] Bergmann O, Bhardwaj RD, Bernard S et al. Science 2009;324:98–102. 19342590 10.1126/science.1164680 PMC2991140

[4] Senyo SE, Steinhauser ML, Pizzimenti CL et al. Nature 2013;493:433–6. 23222518 10.1038/nature11682 PMC3548046

[5] Magadum A, Ding Y, He L et al. Cell Res 2017;27:1002–19. 28621328 10.1038/cr.2017.84 PMC5539351

[6] Mohamed TMA, Ang Y-S, Radzinsky E et al. Cell 2018;173:104–116.e12. 29502971 10.1016/j.cell.2018.02.014 PMC5973786

[7] Nakada Y, Canseco DC, Thet SW et al. Nature 2017;541:222–7. 27798600 10.1038/nature20173

[8] Lopaschuk GD, Karwi QG, Tian R et al. Circ Res 2021;128:1487–513. 33983836 10.1161/CIRCRESAHA.121.318241 PMC8136750

[9] Cardoso AC, Lam NT, Savla JJ et al. Nat Metab 2020;2:167–78. 32617517 PMC7331943

[10] Ajit Magadum NS, Kurian AA, Munir I et al. Circulation 2020. 10.1161/CIRCULATIONAHA.120.047211 PMC7768930 33347328

[11] Li X, Wu F, Günther S et al. Nature 2023;622:619–26. 37758950 10.1038/s41586-023-06585-5 PMC10584682

